# Supplementation of Reduced Gluten Barley Diet with Oral Prolyl Endopeptidase Effectively Abrogates Enteropathy-Associated Changes in Gluten-Sensitive Macaques

**DOI:** 10.3390/nu8070401

**Published:** 2016-06-28

**Authors:** Karol Sestak, Hazel Thwin, Jason Dufour, David X. Liu, Xavier Alvarez, David Laine, Adam Clarke, Anthony Doyle, Pyone P. Aye, James Blanchard, Charles P. Moehs

**Affiliations:** 1 Division of Microbiology, Tulane National Primate Research Center, Covington, LA 70433, USA; hthwin@tulane.edu; 2 PreCliniTria, LLC., Mandeville, LA 70471, USA; 3 Division of Veterinary Medicine, Tulane National Primate Research Center, Covington, LA 70433, USA; jdufour@tulane.edu (J.D.); paye@tulane.edu (P.P.A.); jblanch1@tulane.edu (J.B.); 4 Division of Comparative Pathology, Tulane National Primate Research Center, Covington, LA 70433, USA; dliu1@tulane.edu (D.X.L.); Xavier@tulane.edu (X.A.); 5 TEVA Biologics, Discovery & Development, Sydney, Macquarie Park 2113, NSW, Australia; David.Laine@tevapharm.com (D.L.); Adam.Clarke@tevapharm.com (A.C.); Anthony.Doyle@tevapharm.com (A.D.); 6 Arcadia Biosciences Inc., Seattle, WA 98119, USA

**Keywords:** celiac, gluten, protease, IL-15, oral supplement, gluten-free, rhesus macaque, glutenase, gluten-sensitive enteropathy

## Abstract

Celiac disease (CD) is an autoimmune disorder that affects approximately three million people in the United States. Furthermore, non-celiac gluten sensitivity (NCGS) affects an estimated additional 6% of the population, e.g., 20 million in the U.S. The only effective treatment of CD and NCGS requires complete removal of gluten sources from the diet. While required adherence to a gluten-free diet (GFD) is extremely difficult to accomplish, efforts to develop additional supportive treatments are needed. To facilitate these efforts, we developed a gluten-sensitive (GS) rhesus macaque model to study the effects of novel therapies. Recently reported results from phase one of this project suggest that partial improvement—but not remission—of gluten-induced disease can be accomplished by 100-fold reduction of dietary gluten, i.e., 200 ppm—by replacement of conventional dietary sources of gluten with a mutant, reduced gluten (RG) barley (*lys3a*)-derived source. The main focus of this (phase two) study was to determine if the inflammatory effects of the residual gluten in *lys3a* mutant barley grain could be further reduced by oral supplementation with a prolylendopeptidase (PE). Results reveal that PE supplementation of RG barley diet induces more complete immunological, histopathological and clinical remission than RG barley diet alone. The combined effects of RG barley diet and PE supplementation resulted in a further decrease of inflammatory mediators IFN-γ and TNF secretion by peripheral lymphocytes, as well as decreased plasma anti-gliadin and anti-intestinal tissue transglutaminase (TG2) antibodies, diminished active caspase production in small intestinal mucosa, and eliminated clinical diarrhea—all comparable with a gluten-free diet induced remission. In summary, the beneficial results of a combined RG barley and PE administration in GS macaques may warrant the investigation of similar synergistic approaches.

## 1. Introduction

Any future alternative to the gluten-free diet (GFD) will need to meet a high threshold of safety and efficacy. There is a number of celiac disease (CD) treatments currently in development, including glutenases, enzymes that degrade gluten [1,2,3]. Nevertheless, none of the glutenases are intended to allow celiac patients to deviate from GFD. These are only intended to degrade low quantities of inadvertently ingested gluten. Similarly, low gluten versions of cereals such as barley and wheat are being engineered by transgenic or mutagenic means while most retain sufficient gluten levels to make them incompatible with a GFD [4,5,6]. Several studies have been conducted which focus on the ability of glutenases such as prolylendopeptidase (PE) to help proteolytically degrade those gluten peptides with high content of proline residues that are known to be immunotoxic in celiac and/or gluten-sensitive (GS) patients [1,2,3].

During phase one of this project, we showed that novel varieties of cereal grains with low gluten content such as reduced gluten (RG) barley can have beneficial health effects in GS macaques [7]. In particular, feeding the RG barley diet led to improvement in the villous architecture of the small intestine as well as reduced inflammatory responses in peripheral blood and intestine. Due to the residual amounts of gluten present in RG barley (~1% of that contained in the parental cultivar), feeding of RG barley-derived chow to GS macaques still triggers intermediate inflammatory responses while no such responses could be found in healthy control macaques or GS macaques fed a GFD [7]. Due to the high sensitivity of GS individuals to even minute quantities of dietary gluten, it is important to formulate effective new strategies. Because of its ability to degrade proline-rich residues in immunotoxic gluten peptides [1,2,3,8,9,10,11,12], a PE was added to the RG barley diet, in the current study. The main goal was to determine if such supplementation would lead to a more complete disease remission in GS macaques than that induced by the RG barley diet alone.

## 2. Experimental Section

### 2.1. Ethics Approval

This study was performed with non-human primates. Ethics approval for veterinary procedures was obtained from the Tulane University Animal Care and Use Committee, Animal Welfare Assurance (Ethic approval code: A-4499-01). All procedures were in accordance with the recommendations of the Guide to the Care and Use of Laboratory Animals (NIH) 78-23.

### 2.2. Pre-Screening and Selection of GS Macaques

Pre-screening and identification of candidate macaques for this study (phase two) was done consistent with the methodology described for phase one [7]. After pre-screening, six GS juvenile (<3 years-old) rhesus macaques were assigned to the study based on simultaneous presence of anti-gliadin plasma antibodies (AGA) and anti-tissue transglutaminase (TG2) plasma (IgG) antibodies. Since baseline data with three negative control macaques were already generated under phase one, only GS macaques were used in phase two. All six macaques were free of rhesus-specific enteric pathogens [13].

### 2.3. Diets and Oral Glutenase (PE) Supplement

Three gluten-modified diets were formulated: (1) GFD; (2) conventional (cultivar Bomi) barley diet; and (3) reduced gluten barley (RGB, i.e., Risø 1508 (*lys3a*) derived from Bomi by mutagenesis) diet, consistent with the phase one experiment [7]. The 160–320 g of chow was consumed daily by juvenile macaques. It was estimated that Bomi chow delivered a dose of 2.5–5 g of gluten/day, while the RGB chows delivered approximately 32–64 mg of gluten/day. In order to accelerate progression of disease relapse, Bomi diet was fed to macaques together with a slice of wheat bread per day for each animal (Bomi + B). Based on past experiments [7], each diet was administered for at least one month to induce disease remission (GFD), relapse (Bomi + B), followed by RGB, and RGB orally supplemented with PE (RGB + PE) regimens, to evaluate if RGB + PE regimen induces more complete remission than RGB diet alone. GFD started two weeks after the macaques were assigned to the study, i.e., switched from regular, gluten-containing monkey chow (GD) to GFD. A distinct feature of the phase two experiment was that the RG diet was not only used alone, but also in conjunction with PE oral supplementation, i.e., 580,000 Protease Picomol International (PPI) per animal, consistent with manufacturer’s recommended dose (Tolerase^®^ G, DSM Nutritional Products, Heerlen, The Netherlands). Individual doses of PE (1 g each per day given with chow) were mixed and dissolved in 50 mL of Gatorade every morning and provided using feeding bottles to each of the six macaques for one month. Evaluating whether inclusion of PE into a RGB diet would further ameliorate symptoms of GS in juvenile macaques was the main purpose of this (phase two) study.

### 2.4. Samples and Data Collected

All six macaques were evaluated daily for the symptoms of clinical diarrhea and dehydration ranging between 1 (normal, formed stool), 1.5 (pasty), 2.0 (semi-liquid), 2.5 (liquid), and 3 (liquid, dehydrated). Once every two weeks, animals were bled (5 mL EDTA) and rectally swabbed in order to obtain plasma, peripheral blood mononuclear cells (PBMCs), and gut microbiome samples. At four selected time points, small intestinal biopsies were collected to evaluate the impacts of four dietary regimens (GFD, Bomi + B, RGB and RGB + PE) on intestinal tissue architecture, as described [14,15].

### 2.5. Histopathological Evaluation, AGA, TG2 Antibody Responses and Fluorescent-Activated Cell Sorting (FACS)

Histopathological evaluation of small intestinal biopsies representing each of the four dietary periods (GFD, Bomi + B, RGB and RGB + PE) was done via microscopic evaluation of hematoxylin and eosin (H&E)-stained tissue sections, as described [14]. In addition, AGA and TG2 plasma antibodies were measured every two weeks (July 2015–January 2016). Briefly, pepsin-trypsin digested gliadin was prepared as described [16] and used at a concentration of 20 μg/mL to coat the 96-well plates (Corning, NY, USA). For anti-TG2 test, 10 μg/mL of recombinant human TG2 was used. Antigen-coated plates were washed 3× with 1× PBS, pH 7.4, 0.05% Tween-20 prior to blocking and between all subsequent steps. Plasma samples were diluted 1:1000 in blocking buffer and 100 μL/well was incubated overnight at 4 °C. Secondary, alkaline phosphatase-conjugated antibodies (rabbit anti-monkey IgG, Sigma-Aldrich, St. Louis, MO, USA) were diluted 1:250 in blocking buffer and 100 μL/well was incubated at 3 h at room temperature. Para-Nitrophenylphosphate (pNPP) substrate (Sigma-Aldrich) was used to develop the color reaction in 3 wells, i.e., 3 absorbance values/sample were recorded at 405 nm as described in [16]. The expression of PBMC inflammatory and inhibitory markers—including interferon-gamma (IFN-γ) by CD3+CD8+ T cells, tumor necrosis factor (TNF) by CD20+ B and CD3+CD4+ T cells, and cytotoxic T-lymphocyte-associated protein 4 (CTLA-4 also known as CD152) expression by CD3+CD4+ T cells—was monitored at time points representing the four dietary periods by Fluorescent-Activated Cell Sorting (FACS) [7]. Briefly, PBMCs were stained with a mix of fluorescently-labeled antibodies specific for extracellular antigens first (CD3, CD4, CD8, CD20, and CD152) first, according to the manufacturer’s instructions (BD Pharmingen, San Diego, CA, USA). In order to detect intracellular antigens (TNF and IFN-γ), cells were stimulated with 0.1 μM phorbol miristate acetate (PMA) and 0.5 μg/mL ionomycin (Sigma) and processed according to instructions (BD Pharmingen). Samples were resuspended in BD Stabilizing Fixative (BD Biosciences, San Jose, CA, USA) and data were acquired on FACSAria flow cytometer (BD Biosciences). Data were analyzed by Flowjo software (Tree star, Ashland, OR, USA).

### 2.6. Confocal and Morphometric Evaluation of Small Intestinal Biopsies

Proximal jejunum biopsy tissues collected at time points representing the four dietary periods (GFD, Bomi + B, RGB, and RGB + PE) were used to evaluate gluten-sensitive enteropathy (GSE)-associated changes within the intestinal mucosa. Biopsies were collected and processed as described [14,15]. Briefly, tissues were embedded in paraffin and 7 μm sections were stained first with unconjugated primary antibodies, including: (1) antibodies to tight junction protein Zonula Occludens-1 (ZO-1) as a marker of epithelial integrity; (2) active caspase as a marker of cell apoptosis; and (3) cytokeratin 1 as an epithelial cell marker: (ZO-1, 33-9100, Life Technol. Invitrogen, Waltham, MA, USA; IgA, 617-101-006, Rockland, Inc., Pottstown, PA, USA; Active Caspase, Ab13847, Abcam; Cytokeratin 1, CKLMW 8/18, Biocare Medical; Villin, 2369 S, Cell Signaling Technol. and DAPI nuclear DNA stain, D1306, ThermoFisher Scientific, Waltham, MA, USA). Primary antibodies were followed by appropriate secondary fluorochrome-conjugated antibodies. Confocal microscopy with a Leica TCS SP8 laser scanning confocal microscope system equipped with four lasers, with eight laser lines available, capable of simultaneously collecting information in six channels (five fluorescent and one for differential interference contrast) was used to collect images. Image analysis was performed with Volocity software (version 6.3, PerkinElmer, Waltham, MA, USA) to count the apoptotic cells on a software-generated grid (Supplementary Materials Figure S1). Epithelium and lamina propria from biopsied animals were evaluated for the extent of apoptosis.

### 2.7. Immunohistochemistry of IL-15 in Rhesus Small Intestine

To develop the immunohistochemistry procedure, antigen-positive and negative control samples were generated. Expi 293F (a derivative of HEK-293) cell lines were transiently transfected with either rhesus IL-15 (GenBank, U19843.1) or mock plasmid according to manufacturer’s instructions (Life Technologies, ExpiFectamine™ 293 Transfection Kit A14526, Carlsbad, CA, USA). After 48 hours, cells were harvested and assessed for expression of rhesus IL-15 by flow cytometry on live cells or on 4% paraformaldehyde (PFA)-fixed cells using anti-human IL-15 antibody (R & D Systems, MAB647) and an IgG1k isotype control (Abcam, AB18443) mouse monoclonal antibody. It was established that MAB647 antibody reacts with rhesus IL-15 (Supplementary Materials Figure S2). Transfected PFA-fixed cells were then used to identify optimal conditions for the positive immunohistochemistry tissue staining including antibody concentrations that would result in minimal background.

To perform small intestinal tissue analysis, MAB647 and an IgG1k isotype control antibody were used as the primary antibodies at 20 μg/mL on 4% PFA-fixed small intestine tissue samples from GS and healthy control rhesus monkeys. The detection system consisted of anti-mouse secondary (Vector, BA-2000) and ABC-Peroxidase kit (Vector, PK-4000) with a DAB + chromagen substrate kit (DAKO, K3468), yielding the brown-colored deposit. Slides were imaged with a DVC 1310C digital camera coupled to a Nikon microscope. The entire immunohistochemistry procedure was also performed on an adjacent section of the 4% PFA-fixed control tissue in the absence of primary antibody to serve as a negative control. Brown color intensity was recorded on a 0–4 scale (0 = negative, 1 = blush, 2 = faint, 3 = moderate, 4 = strong).

### 2.8. Statistical Analysis

Graphical representation and statistical analysis of the cytokine-producing, apoptotic cell, CTLA-4 data and clinical diarrhea scores were performed using the GraphPad Prism 6.0 (GraphPad Software, San Diego, CA, USA). Comparisons between the time-points corresponding to each diet were done for each measurement (plasma AGA and TG2 antibodies, clinical diarrhea scores, and cytokine/apoptotic cells) by Mann-Whitney U-test. Values of *p* < 0.05 were considered statistically significant.

## 3. Results

### 3.1. Peripheral AGA and TG2 Antibody Responses

**Figure 1:**
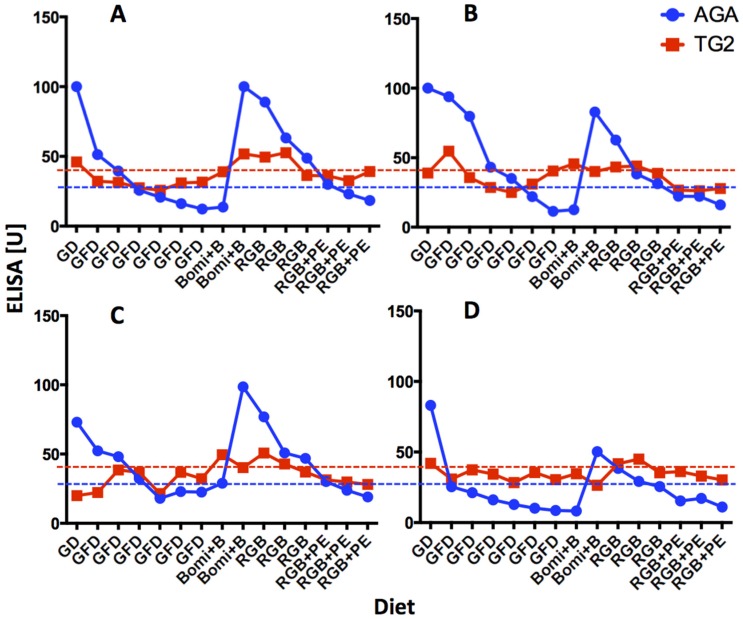
Anti-gliadin antibodies (AGA) and anti-intestinal tissue transglutaminase (TG2) plasma antibodies in four gluten-sensitive (GS) rhesus macaques (**A**–**D**). Gluten-modified diets (GD, GFD, Bomi + B, RGB and RGB + PE) that were used to feed the macaques are indicated. Individual time points represent two-week intervals. Blue dashed line represents AGA baseline, i.e., 25 ELISA units while red dashed line represents TG2 antibody baseline, i.e., 40 units. Values elevated above these lines were significantly greater (*p* < 0.05) than values generated with plasmas from healthy, normal macaques.

Withdrawal of dietary gluten (GFD) resulted in complete remission of plasma AGA and TG2 antibody levels within one month (Figure 1). Elevated AGA and TG2 antibody responses reflected administration and re-introduction of dietary gluten (GD and Bomi + B diets, respectively) while its removal (GFD) or replacement with RGB diet were followed by lowered levels of both antibodies.

Notably, decrease in AGA and TG2 antibodies continued after the introduction of PE into RGB diet (RGB + PE). Only upon RGB + PE diet administration did the AGA and TG2 antibodies decline below or close to baseline levels. There were noticeable similarities in the dynamics of AGA and TG2 antibody formation in studied GS macaques but also differences in magnitude of these responses, likely attributed to heterogeneous *Mamu* II backgrounds of the GS macaques used. As previously reported, none of the healthy controls used in phase one of this study developed any AGA or TG2 antibody serum responses [7].

### 3.2. Clinical Diarrhea Scores

**Figure 2:**
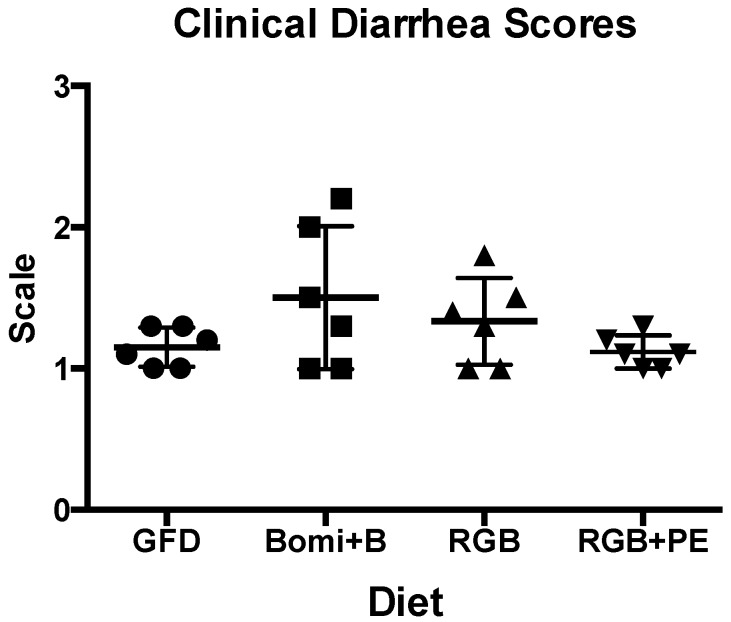
Clinical diarrhea scores generated with GS macaques fed gluten-modified diets (GFD, Bomi + B, RGB and RGB + PE) are shown.

Clinical diarrhea scores (Figure 2) reflected plasma AGA and to a lesser extent TG2 antibody levels following the administration of the four experimental diets (Figure 1 and Figure 2). Despite the fact that the Bomi + B diet was fed for no longer than one month, re-introduction of barley- and wheat-derived gluten in this diet caused clinical diarrhea in GS macaques. The significantly (*p* < 0.05) elevated clinical diarrhea scores of Bomi + B diet fed macaques above those of GFD fed macaques were suggestive of progression towards more severe diarrhea in the scenario where Bomi + B diet would not be replaced by RGB diet (Figure 2). Once Bomi + B diet was replaced by RGB and later followed by RGB + PE diets, clinical diarrhea scores returned to normal, healthy animal levels within two months.

### 3.3. Rhesus Macaque Small Intestinal Tissue Architecture

**Figure 3:**
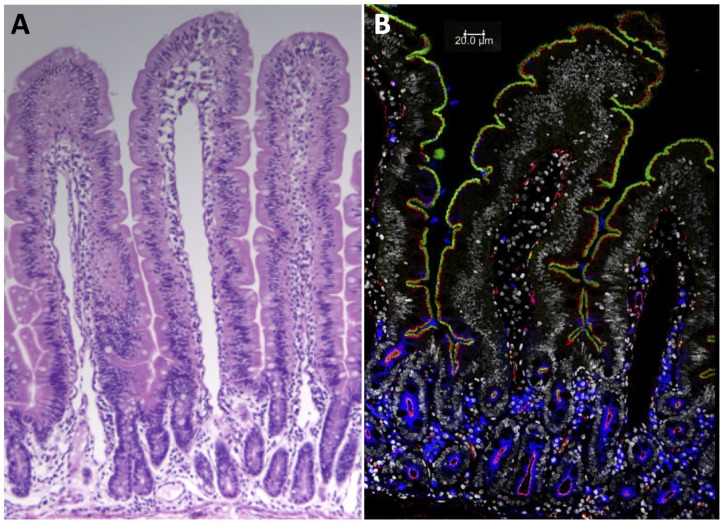
H & E staining of jejunum from a GS macaque while on GFD reveals normal-range intestinal architecture, magnification 10× (**A**); Four-color confocal microscopy of jejunum from another animal on GFD (**B**) shows undisrupted continuity of villin (**green**) and tight junction protein ZO-1 staining (**red**). Abundant IgA-positive B cells are seen in the subepithelium (**blue**). Gray = nuclear DNA.

H & E staining of small intestinal biopsy tissues from juvenile GS macaques while on GFD revealed normal tissue architecture, without villous atrophy or extensive lymphocytic infiltrations of lamina propria (Figure 3A), unlike the considerable GSE that is seen in GS macaques on a long-term GD [14,16].

Typically, an advanced GSE is in macaques accompanied with discontinuous expression of tight junction proteins such as ZO-1, resulting in a compromised epithelial barrier function [15,17] but this was not seen in juvenile macaques fed Bomi + B diet. The IgA-positive B cells were observed in lamina propria of GS macaques regardless of GFD treatment (Figure 3B).

### 3.4. Gluten Diet-Dependent Apoptotic Changes in Small Intestine

**Figure 4:**
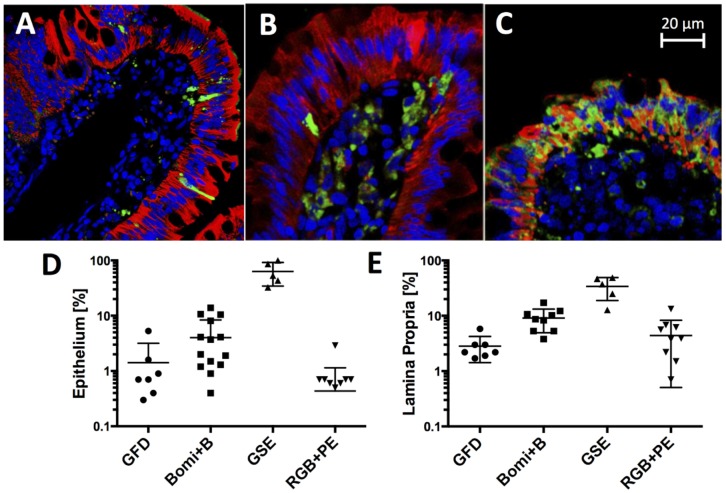
The combination of cytokeratin 1 (**red** = epithelial cells), active caspase (**green** = cells undergoing apoptosis) and diamino-2-phenylindole (DAPI, **blue** = nuclear DNA) antibodies was used to examine the diet-induced changes within intestinal mucosa. Jejunum from juvenile GS macaques on gluten-free diet (GFD) shows prominent red staining corresponding to epithelial cells with very few of the green cells (**A**); Jejunum from macaque on Bomi + B diet shows an increased number of green/apoptotic cells positive for active caspase inside the lamina propria (LP) (**B**); Control jejunum tissue from an adult macaque with gluten-sensitive enteropathy (GSE) exhibits high % of epithelial apoptotic cells (**C**); Charts reflecting the proportions (%) of apoptotic cells inside the epithelium (**D**) and LP (**E**) of GS macaques fed dietary gluten-modified diets in this study plus the control GSE macaques fed regular monkey (gluten-containing) chow are shown.

In order to study histopathological changes on a cellular level, confocal microscopy of small intestinal biopsies was used (Figure 4). Epithelial and subepithelial (lamina propria, i.e., LP) cells that were positive for active caspase were designated as “apoptotic” and their proportions (%) from total epithelial and LP cells were recorded. The highest proportions of apoptotic cells were found in historical GSE controls, i.e., adult rhesus macaques with CD-like symptoms that were not part of this study except for use as comparisons. Both epithelial and LP apoptotic cell counts from GSE controls were significantly higher (*p* < 0.001) than those generated with juvenile GS macaques, regardless of the GFD, RGB + G, or Bomi + B diets (Figure 4A–E). The highest proportions of apoptotic cells in juvenile GS macaques were found in biopsies taken while animals were fed Bomi + B diet. After the switch from Bomi + B to RGB diets, the numbers of apoptotic cells started to decline. After one month on RGB diet, followed by one month on RGB + PE diet, apoptotic cell counts decreased to levels comparable with those ascribed to GFD (Figure 4D,E).

It is important to emphasize that differences between counts of apoptotic cells ascribed to Bomi + B vs. RGB + PE diets were significant (*p* < 0.05) for both epithelial and LP counts although not as robust as those observed with GSE controls (*p* < 0.001, Figure 4D,E). In summary, it was observed that after being placed on RGB + PE diet, juvenile GS macaques lowered their numbers of apoptotic enterocytes to levels similar to those associated with a GFD.

### 3.5. IL-15 Expression in Small Intestine

**Figure 5:**
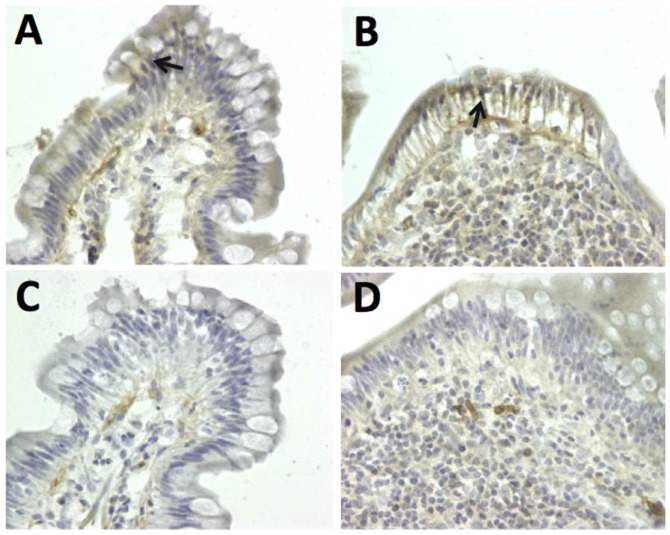
Jejunum biopsies collected from GS macaques on Bomi + B diet show an increased IL-15 staining, indicated by arrows (**A**,**B**) while lower intensity of such signal was detected in tissues stained with unrelated, isotype-matched antibodies or in healthy control biopsies (**C**,**D**), magnification 40×.

Due to emerging evidence regarding the key role of IL-15 in NKT-cell-mediated pathogenesis of CD/GSE [18,19,20], selected jejunum biopsies from three GS and one healthy control macaques fed Bomi + B diets were evaluated for the presence of IL-15. Biopsies from GS macaques showed prominent IL-15 staining (Figure 5A,B) while lower intensity (but not completely negative) of such signal was detected also in tissues stained with unrelated, isotype-matched antibodies as well as healthy control jejunum (Figure 5C,D).

### 3.6. Expression of Pro- and Anti-Inflammatory Mediators by PBMCs

**Figure 6:**
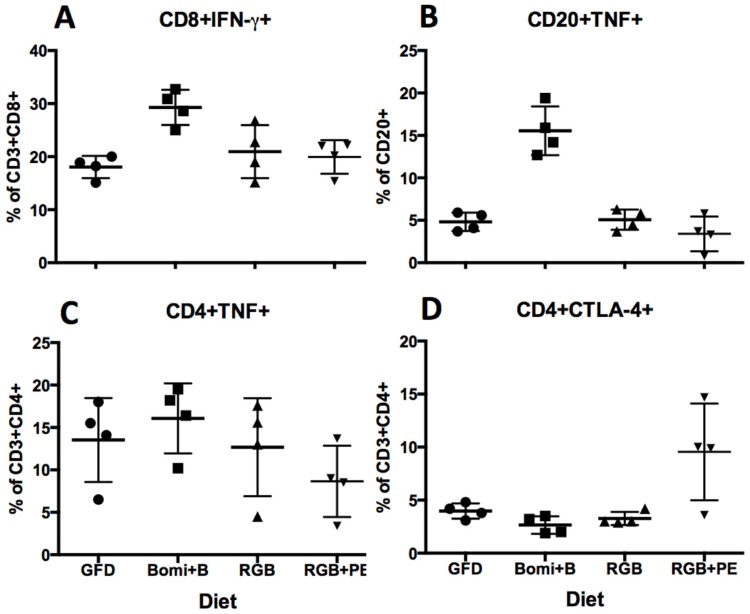
Proportions of pro- and anti-inflammatory mediator expressions by peripheral blood mononuclear cells (PBMCs) following the administration of gluten-modified diets are shown. Significant differences in IFN-γ production between the GFD vs. Bomi + B fed macaques (*p* = 0.006) were found (**A**). Bomi + B vs. RGB or RGB + PE fed macaques also differed significantly (*p* < 0.05) in IFN-γ production (**A**); A similar but more robust difference (*p* < 0.0001) between the Bomi + B fed macaques and other diets was found in the case of TNF production by CD20 + B cells (**B**); Non-significant trends for differences were found in the case of TNF production by CD4 + T cells (**C**) while an increase in expression of anti-inflammatory CTLA-4 (CD152) by CD4 + T cells (*p* < 0.05) was observed after the macaques were placed on RGB + PE diet (**D**). Time intervals between indicated measurements correspond to four weeks.

Proportions of IFN-γ, TNF and CTLA-4 inflammation-regulatory molecules—expressed by peripheral blood lymphocytes—were measured by multi-color FACS. Significant and consistent increases in expression of IFN-γ by CD3 + CD8 + T cells and TNF by CD20 + B cells were found in Bomi + B diet-fed GS macaques (Figure 6A,B). Only a non-significant trend for increased expression of TNF by CD3 + CD4 + T cells was detected following the administration of Bomi + B diet (Figure 6C and Supplementary Materials Figure S3).

An increase (*p* < 0.05) in expression of anti-inflammatory CTLA-4 (CD152) molecule by CD3 + CD4 + T cells was revealed upon introduction of RGB + PE regimen (Figure 6D). Taken together, these findings suggest that several pro-inflammatory changes take place in GS juvenile macaques upon introduction of dietary gluten. Notably, these changes can be reversed upon introduction of RGB + PE diet—the effects of which are comparable with the effects of a GFD.

## 4. Discussion

In order to extend the findings from our recently completed phase one study and to evaluate if inclusion of oral glutenase (*Aspergillus niger*-derived prolyl endopeptidase, i.e., PE) into a reduced-gluten RGB diet will further ameliorate symptoms of GS in juvenile macaques, we conducted this (phase two) study. In accord with phase one, despite its beneficial effects, RGB diet alone was not sufficient to eliminate symptoms of GS in macaques, most likely due to residual quantities of dietary gluten (200 ppm) in RGB chow [7]. Consumption of RGB diet alone in GS macaques is characterized by intermediate levels of TNF and IFN-γ production by peripheral lymphocytes, low-grade intestinal inflammation, and border-line AGA (IgG) plasma levels. Our hypothesis was that these effects would further be abrogated upon inclusion of PE oral glutenase supplement.

Past attempts to utilize PE as oral therapy in celiac patients yielded promising results. The initial report that suggested the applicability of a PE in treatment of CD dates back to 2002 [8]. Since then, several studies—including clinical trials—have been conducted, which have focused on the ability of PEs to proteolytically degrade gluten, which comprises peptides with hard-to-digest, immunotoxic sequences featuring a high proline content [1,2,3]. Some unanswered questions still remain regarding the delivery of oral PE supplement can avoid the proteolytic degradation during gastric passage while ensuring the optimal dosing in small intestine [3,10]. Regardless, PE remains the only digestive supplement capable of degrading most of the gluten-derived immunotoxic epitopes [11]. Due to their biological similarities with human celiac patients, GS rhesus macaques were used to evaluate combined effects of PE and barley-derived, reduced-gluten diet. Juvenile GS macaques that did not yet have the fully-developed form of the disease (GSE) were used to assure the prompt and effective response upon administration of RGB + PE diet and treatment, as opposed to some adult rhesus macaques with severe GSE where dietary gluten withdrawal and treatment takes a much longer time to have an effect [16]. Variable levels of villous atrophy and lymphocytic infiltration of lamina propria were observed in juvenile macaques consuming Bomi + B diet [7]. In contrast to the severe GSE that develops as a consequence of life-long dietary gluten consumption in some—but not all—GS adult rhesus macaques [14,16], one month’s administration of Bomi + B diet to juvenile GS macaques did not result in morphologically distinguishable, severe GSE but only mild enteritis. A commercially available form of PE was used (Tolerase G^®^, DSM, Heerlen, The Netherlands), following the manufacturer’s recommended dose and route of administration to ameliorate the symptoms of GS.

In addition to established hallmarks of GS in macaques such as AGA and TG2 serum antibodies, chronic diarrhea, enteritis, compromised epithelial integrity (measured by ZO-1 tight junction protein expression), and/or GSE, as well as increased expression of IFN-γ by peripheral and/or intestinal lymphocytes, the following measurements were examined in this study: (1) Quantitative analysis of enteric (small intestine) apoptosis; and (2) Qualitative evaluation of IL-15 production in small intestine. It was demonstrated that apoptosis plays a role in CD and diabetes mellitus patients in enterocyte destruction, preceding the development of villous atrophy (VA) [21,22,23]. It was found that cytokeratin 18 caspase-cleaved fragment, granzyme B, and other factors play roles in this process. It has also been reported that down-regulation of apoptotic inhibitors in patients with refractory CD induces further pro-apoptotic effects [22]. Although severe VA can develop in adult rhesus macaques with GS, it was not yet fully evident in this study’s juvenile animals. Therefore, a morphometric evaluation of enteric mucosa was conducted, hypothesizing that reduced-gluten diets and PE treatment would have an impact on epithelial enterocytes’ apoptosis. A high resolution, multi-laser Leica confocal microscope equipped with Volocity cell imaging software was used for this purpose. Interestingly, apoptosis not only took place in intestinal epithelium upon introduction of dietary gluten (Bomi + B diet) but its extent was significantly different between GS macaques fed Bomi + B and RGB + PE diets. This result alone represents the most significant finding from our study. It illustrates that enteropathy-associated changes that occur in intestinal mucosa prior to development of severe GSE are reversible, and can be achieved not only upon administration of GFD but also by RGB + PE diet. These findings provide guidance for further research involving gluten-modified diets and also illustrate the usefulness of the GS rhesus model in preclinical research.

Evaluation of IL-15 production within the small intestine of GS macaques confirmed the anticipated increased presence of this key regulator of CD immunopathology [20]. Clearly, IL-15 production represents an entirely different (NKT-cell mediated epithelial cell destruction) mechanism of GSE pathogenesis than that linked with intestinal apoptosis. Nonetheless, elevated levels of IL-15 in GS macaques corroborate the overall validity of the GS macaque model and suggest that more than one mechanism contributes to GSE pathogenesis. An unanticipated increase in CTLA-4 expression by peripheral CD4 + T cells following the supplementation of RGB diet with PE was also observed (Figure 6D). Considering the beneficial role CTLA-4 was suggested to play in down-regulation of autoimmune diseases including CD [24,25], this result deserves further examination. Intestinal apoptosis, IL-15, and CTL-4 traits of CD pathogenesis should be investigated on a molecular level in future studies. The rhesus GS model is a “natural-disease model” that closely resembles the biology and pathogenesis of human CD and does not require genetic manipulation of its host. Future studies might also be conducted with inhibitors of intestinal apoptosis and/or NKT-cell mediated autoimmunity as alternative treatments of CD.

As our study was underway, a publication appeared [26] indicating that additional gluten-reducing mutations had been combined with the *lys3a* mutant present in the RGB used in the present study to produce an ultra-low gluten barley (ULGB). This ULGB has been used to brew beer that can be classified as gluten-free (containing less than 20 ppm gluten). Presumably at this low level of gluten, beer made with ULGB may be safe for celiac patients, although this remains to be rigorously evaluated. Treatments such as the glutenase Tolerase^®^ G would likely provide an additional margin of safety. Efforts to produce an analogous ultra-low gluten wheat are less likely to be successful both because of the more complex genetics of tetraploid pasta and hexaploid bread wheat and because of the constraints imposed by the required functional properties of the wheat, in which the gluten proteins play a major role.

## 5. Conclusions

Our recently reported results suggest that partial improvement but not complete remission of gluten-induced disease can be accomplished by 100-fold reduction of dietary gluten—by replacement of conventional dietary sources with barley-derived, reduced gluten (*lys3a* barley) source. The main focus of this study was to determine if the inflammatory effects of leftover gluten in the RGB grain could be further reduced by oral supplementation with PE. Our results show that PE supplementation of RGB diet induces more complete immunological, histopathological, and clinical remission than RGB diet alone. The beneficial effects of the RGB + PE treatment on GS juvenile macaques were comparable with those of the GFD. These findings provide guidance for further research involving gluten-modified diet alternatives.

## Supplementary Materials

The following are available online at http://www.mdpi.com/2072-6643/8/7/401/s1, Figure S1: Confocal microscopy of paraffin-fixed tissue sections labeled with fluorescein-conjugated antibodies to active caspase (apoptotic cells = green), cytokeratin 1 (epithelial cells = red) and nuclear DNA (blue) was used in conjunction with 6.3 Volocity 3D cell imaging software (PerkinElmer) to enumerate the apoptotic cells on a software-generated grid; Figure S2: Negative mock (A) and positive control, human and rhesus IL-15 transfected Expi 293F cells, were used to optimize the IL-15 staining; Figure S3: Peripheral blood populations of CD3 + T and CD20 + B lymphocytes (A) were evaluated for the production of regulatory and inflammatory cytokines including IFN-γ and TNF. CD3 + T lymphocytes were subdivided into CD4 + and CD8 + cells (B).
