# Supplementary material for: Supplementation of Reduced Gluten Barley Diet with Oral Prolyl Endopeptidase Effectively Abrogates Enteropathy-Associated Changes in Gluten-Sensitive Macaques
Source: Nutrients. 2016 Jun 28;8(7):401. doi: 10.3390/nu8070401 (PMC4963877; doi:10.3390/nu8070401)
Supplement: Supplementary file 1 [file nutrients-08-00401-s001.docx]

Supplementary Materials: Supplementation of Reduced Gluten Barley Diet with Oral Prolyl Endopeptidase Effectively Abrogates Enteropathy-Associated Changes in Gluten-Sensitive Macaques

Karol Sestak, Hazel Thwin, Jason Dufour, David X. Liu, Xavier Alvarez, David Laine,
Adam Clarke, Anthony Doyle, Pyone P. Aye, James Blanchard and Charles P. Moehs


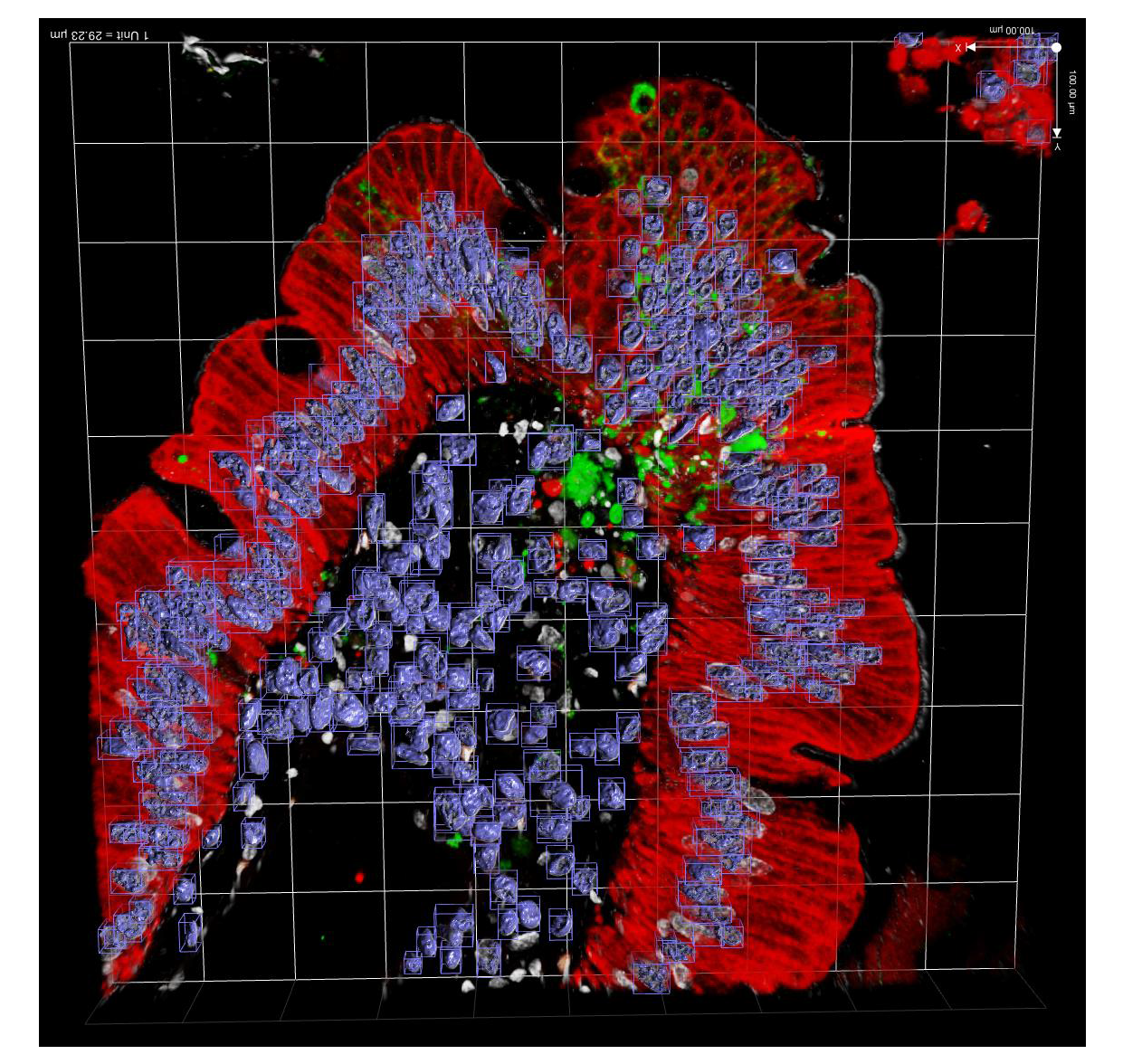


**Figure S1.** Confocal microscopy of paraffin-fixed tissue sections labeled with fluorescein-conjugated antibodies to active caspase (apoptotic cells = **green**), cytokeratin 1 (epithelial cells = **red**) and nuclear DNA (**blue**) was used in conjunction with 6.3 Volocity 3D cell imaging software (PerkinElmer) to enumerate the apoptotic cells on a software-generated grid.


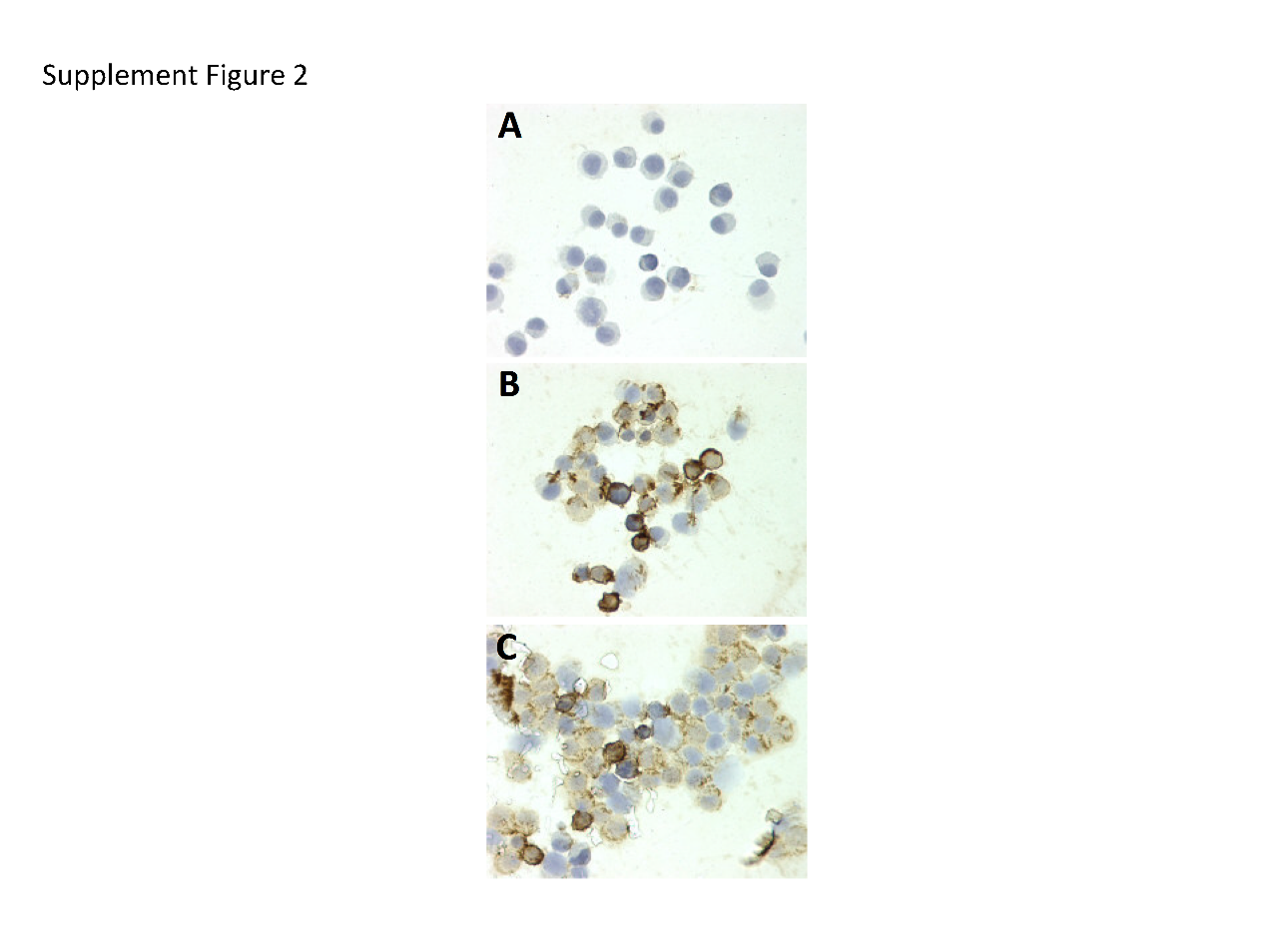


**Figure S2.** Negative mock (**A**) and positive control, human and rhesus IL-15 transfected Expi 293F cells, were used to optimize the IL-15 staining. As shown by positive (**brown** color) reaction with anti-IL-15 antibodies, cells were successfully transfected with both: human (**B**) and rhesus (**C**) IL-15.


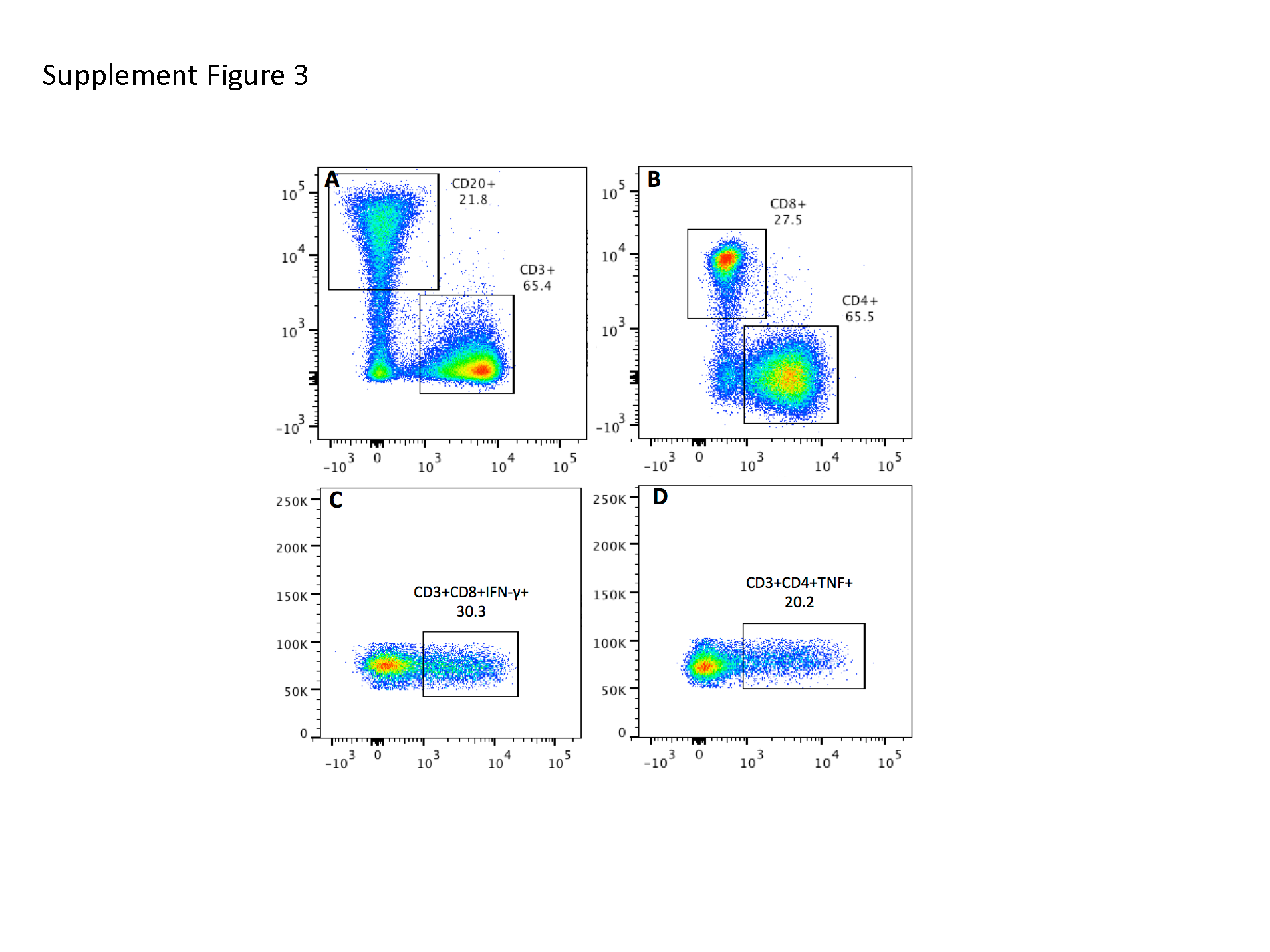


**Figure S3.** Peripheral blood populations of CD3 + T and CD20 + B lymphocytes (**A**) were evaluated for the production of regulatory and inflammatory cytokines including IFN-γ and TNF; CD3 + T lymphocytes were subdivided into CD4 + and CD8 + cells (**B**); After feeding the Bomi + B diet for four weeks, selected macaque responses are shown for IFN-γ production by CD3 + CD8 + T cells (**C**) and TNF production by CD3 + CD4 + T cells (**D**).
